# Regulatory T Cells in Patients with Idiopathic Thrombocytopenic Purpura

**DOI:** 10.4274/tjh.2015.0335

**Published:** 2016-05-16

**Authors:** Alev Akyol Erikçi, Bülent Karagöz, Oğuz Bilgi

**Affiliations:** 1 Gülhane Military Medical Academy, Haydarpaşa Training and Research Hospital, Clinic of Hematology, İstanbul, Turkey

**Keywords:** Idiopathic thrombocytopenic purpura, Regulatory T cells

## Abstract

**Objective:**

Immune thrombocytopenic purpura (ITP) is an immune-mediated bleeding disorder in which platelets are opsonized by autoantibodies and destroyed by an Fc receptor-mediated phagocytosis by the reticuloendothelial system within the spleen. Autoimmune processes are also considered in the pathogenesis of this disorder. CD4+CD25+FoxP3+ regulatory T (Treg) cells and CD8+CD28- Treg cells have roles in autoimmune diseases. We investigated these regulatory cells in ITP patients.

**Materials and Methods:**

We included 22 ITP patients and 16 age-matched healthy subjects. CD4+CD25+FoxP3+ Treg cells and CD8+CD28- cells were investigated by three-color flow cytometry. The ratios of these cell populations to total lymphocytes were calculated. Statistical analysis was carried out with the Mann-Whitney U test.

**Results:**

CD4+CD25+ Treg cells were 9.69±3.70% and 12.99±5.58% in patients with ITP and controls, respectively. CD4+CD25highFoxP3+ cells were 27.72±19.74% and 27.55±23.98% in ITP patients and controls, respectively. The percentages of both of these cell types were not statistically significant when compared to the control group.

**Conclusion:**

We did not find any differences in ratios of CD4+CD25+FoxP3+ Treg cells or CD8+CD28- T cells in lymphocytes between patients and healthy subjects. We conclude that these circulatory cells are not different in ITP, but further studies are needed to explore the putative roles of these regulatory cells.

## Abstract (tr)

**Amaç:**

İmmün trombositopenik purpura (İTP) trombositlerin otoantikorlar tarafından opsonize edildiği ve retiküloendotelyal sistem tarafından Fc reseptör aracılı fagositoz ile dalakta yıkıldığı immün kaynaklı bir kanama bozukluğudur. Bu bozukluğun patogenezinde otoimmün süreçler de sorumlu tutulmaktadır. CD4+CD25+Foxp3+ regulatuvar T (Treg) hücreleri ve CD8+CD28- Treg hücreler otoimmün hastalıklarda rol oynamaktadır. Çalışmamızda İTP’li hastalarda bu regülatuvar hücreleri araştırdık.

**Gereç ve Yöntemler:**

İTP’li 22 hasta ile yaş uyumlu 16 sağlıklı birey dahil edildi. CD4+CD25+Foxp3+ Treg hücreler ve CD8+CD28- hücreler üç renkli akım sitometri ile çalışıldı. Bu hücre popülasyonunun tüm lenfositlere oranı hesaplanmıştır. İstatiktiksel değerlendirmede Mann-Whitney U testi kullanılmıştır.

**Bulgular:**

CD4+CD25+ Treg hücreler İTP’de ve kontrol grubunda %9,69±3,70 ve %12,99±5,58 saptandılar. CD4+CD25 yüksek FoxP3+ hücreler ise İTP’de ve kontrol grubunda %27,72±19,74 ve %27,55±23,9 olarak saptandı. Her iki hücre tipi de kontrol grubu ile karşılaştırıldığında istatiktiksel olarak anlamlı bulunmamıştır.

**Sonuç:**

Lenfositlerdeki CD4+CD25+Foxp3+ Treg hücreler ve CD8+CD28- T hücrelerdeki oranlarında fark bulamadık. Biz çalışmamızda İTP’de dolaşan regulatuvar hücrelerde fark bulamadık ama daha geniş kapsamlı çalışmalara ihtiyaç vardır.

## INTRODUCTION

Immune thrombocytopenia (ITP) is an autoimmune bleeding disorder in association with increased platelet destruction and impaired platelet production. It is mediated by IgG antiplatelet autoantibodies in which the targets are platelet membrane glycoproteins (GPs), such as GPIIb/IIIa and GPIb/IX. CD4+CD25+ regulatory T (Treg) cells and CD8+CD28- T lymphocytes have major roles in self-tolerance. To maintain the immune tolerance and to prevent autoimmune disease, CD4+CD25+FoxP3+ Treg cells, CD4+ T cells with high expression of CD25, and transcription factor forkhead box P3 (FoxP3), also referred to as FoxP3 regulatory T cells, play an important role. Treg cells account for approximately 5% of circulating CD4+ T cells. Decreased numbers of Treg cells have been reported in patients with various autoimmune diseases, including ITP, rheumatoid arthritis, and systemic lupus erythematosus [1,2,3,4,5].

In the case of Treg deficiency, peripheral tolerance can fail, leading to the development of autoimmunity. The purpose of this study was to evaluate Treg cells in previously untreated newly diagnosed ITP cases.

## MATERIALS AND METHODS

### Flow Cytometry

Peripheral blood samples were obtained and studied while still fresh. Flow cytometry was used to count CD4+CD25+ Treg cells and CD8+CD28- suppressive cells. Flow cytometry was performed on a Becton Dickinson FACSCalibur. Data were obtained and analyzed using CellQuest software.

### Monoclonal Antibodies

Antihuman monoclonal antibodies conjugated with fluorochromes and appropriate isotype controls were used: fluorescein isothiocyanate (FITC) conjugated anti-CD28 (BD Pharmingen Catalog No: 555728), anti-CD4 (Caltag Laboratories Catalog No: MHCD0401), phycoerythrin-cyanine 5 (PC5) conjugated anti-CD8 (eBioscience Catalog No: 15-0088), anti-CD25 (BD Pharmingen Catalog No: 555433), and phycoerythrin (PE) conjugated anti-FoxP3 (eBioscience Catalog No: 12-4776).

### Cell Preparation and Surface Staining

Human peripheral blood mononuclear cells were isolated using Histopaque (Sigma Catalog No: 1077) gradient centrifugation. Aliquots of 100 µL were transferred to polypropylene test tubes (12x75 mm; BD Bioscience Catalog No: 352052) and 20 µL of conjugated monoclonal antibodies or isotype controls was added to each tube. Flow cytometric analysis was performed by BD FACSCalibur after the appropriate staining protocol.

### FoxP3 Staining

CD4 and CD25 surface staining was carried out. The CD4+CD25 tube was then washed with cold PBS and resuspended, 1 mL of freshly prepared fixation/permeabilization working solution was added, and the tube was incubated at 4 °C for 30-60 min in the dark and washed twice by adding 2 mL of 1X permeabilization buffer. Next, 20 µL of PE conjugated antihuman FoxP3 antibody in 1X permeabilization buffer was added and the tube was incubated at 4 °C for 30 min in the dark. Washing was repeated twice with 2 mL of 1X permeabilization buffer. After resuspension, analysis was performed by flow cytometry.

### Analysis

CD8+CD28- cell percentages were evaluated using anti-CD28/anti-CD8 double staining in lymphocyte-gated cells. CD8+CD28- cells, CD8+CD28+ cells, and the ratio of these cells were calculated.

Anti-CD4/anti-FoxP3/anti-CD25 triple staining was uved for CD4+CD25+ Treg cell counts. CD4+CD25high lymphocytes were gated and then CD4+CD25highFoxP3+ cells were calculated in CD4/FoxP3 histograms.

### Statistical Analysis

Statistical analysis was performed using SPSS. The Mann-Whitney U test was used to investigate immunological parameters of ITP patients and for comparisons with data of healthy subjects.

## RESULTS

We enrolled 22 previously untreated patients newly diagnosed with ITP (19 males, 3 females) and 16 age-matched controls (13 males, 3 females). All of the patients were admitted to our outpatient clinic. Thrombocytopenia was newly detected and they had received no previous treatment. The patients were investigated for possible causes of thrombocytopenia. Viral serology and other underlying autoimmune diseases were screened. Demographic findings are illustrated in Table 1. We performed bone marrow aspiration and biopsy in the relatively elderly patients (patients numbers 5, 9, and 17). No pathological findings such as dysplasia were detected. Findings were consistent with ITP, including normal or increased megakaryocytes.

CD4+CD25+ Treg cells and CD4+CD25highFoxP3+ cells were calculated in lymphocytes. CD4+CD25+ Treg cells were 9.69±3.70% and 12.99±5.58% in patients with ITP and controls, respectively. CD4+CD25highFoxP3+ cells were 27.72±19.74% and 27.55±23.98% in ITP patients and controls, respectively. Both of these cell counts were not statistically different between groups.

We also detected no statistically significant difference in CD8+CD28- suppressor cells between ITP patients and controls (12.50±9.40% and 11.77±4.64%, respectively).

## DISCUSSION

Treg cells suppress effector T cell activation, which leads to induction of immune tolerance [6]. For this reason it is assumed that failure of the regulatory T cell system may induce autoimmunity [7,8,9].

There are increasing numbers of studies demonstrating that decreased frequency of Treg cells has a role in ITP. Liu et al. reported that the percentage of Treg cells was significantly decreased in ITP patients with active disease in which no remission was achieved [10]. Sakakura et al. reported variations in Treg amounts according to platelet counts. In patients with low platelet counts there was no reduction in the percentage of Treg cells when compared to those with platelet counts over 100,000/µL [11]. In the study by Yu et al., defective circulating CD25 Treg cells were detected in patients with chronic ITP [12].

However, there are also studies that failed to detect any differences in Treg frequencies of patients with ITP compared to healthy controls [13,14].

Similar to our results, Mazzucco et al. detected no significant difference between Treg cell and platelet counts in patients with ITP and the control group [15].

In our study we investigated previously untreated newly diagnosed ITP patients. We detected no significant difference in Treg cell frequencies in ITP patients and controls. We think that further studies are needed to explore the putative roles of these regulatory cells, especially in terms of long-term follow-ups and response to treatments.

## Ethics

Informed Consent: It was taken.

## Figures and Tables

**Table 1:**
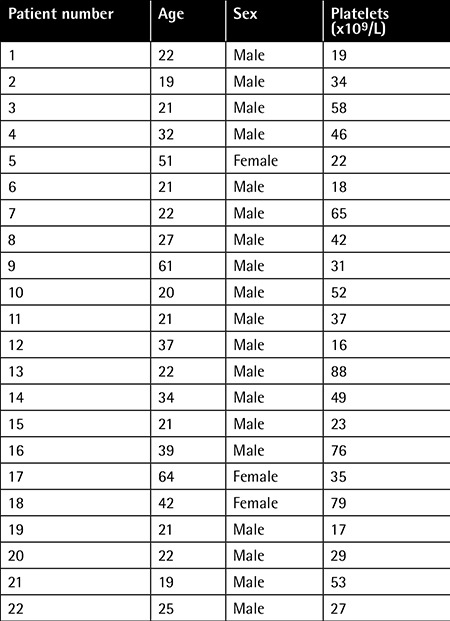
Patients’ characteristics

## References

[1] Cines DB, Blanchette VS (2002). Immune thrombocytopenic purpura. N Engl J Med.

[2] Semple JW, Freedman J (1991). Increased antiplatelet T helper lymphocyte reactivity in patients with autoimmune thrombocytopenia. Blood.

[3] Kuwana M, Kaburaki J, Ikeda Y (1998). Autoreactive T cells to platelet GPIIb-IIIa in immune thrombocytopenic purpura: role in production of anti-platelet autoantibody. J Clin Invest.

[4] Ogawara H, Handa H, Morita K, Hayakawa M, Kojima J, Amagai H, Tsumita Y, Kaneko Y, Tsukamoto N, Nojima Y, Murakami H (2003). High Th1/Th2 ratio in patients with chronic idiopathic thrombocytopenic purpura. Eur J Haematol.

[5] Semple JW, Milev Y, Cosgrave D, Mody M, Hornstein A, Blanchette V, Freedman J (1996). Differences in serum cytokine levels in acute and chronic autoimmune thrombocytopenic purpura: relationship to platelet phenotype and antiplatelet T-cell reactivity. Blood.

[6] Itoh M, Takahashi T, Sakaguchi N, Kuniyasu Y, Shimizu J, Otsuka F, Sakaguchi S (1999). Thymus and autoimmunity: production of CD25+CD4+ naturally anergic and suppressive T cells as a key function of the thymus in maintaining immunologic self-tolerance. J Immunol.

[7] Nugent DJ (2006). Immune thrombocytopenic purpura of childhood. Hematology.

[8] Cruvinel WM, Mesquita D, Araujo JAP, Salmazi KC, Kallas EG, Andrade LEC (2008). Natural regulatory T cells in rheumatic diseases. Rev Bras Reumatol.

[9] Sakaguchi S, Ono M, Setoguchi R, Yagi H, Hori S, Fehervari Z, Shimizu J, Takahashi T, Nomura T (2006). Foxp3+CD25+CD4+ natural regulatory T cells in dominant self-tolerance and autoimmune disease. Immunol Rev.

[10] Liu B, Zhao H, Poon MC, Han Z, Gu D, Xu M, Jia H, Yang R, Han ZC (2007). Abnormality of CD4+CD25+ regulatory T cells in idiopathic thrombocytopenic purpura. Eur J Haematol.

[11] Sakakura M, Wada H, Tawara I, Nobori T, Sugiyama T, Sagawa N, Shiku H (2007). Reduced Cd4+Cd25+ T cells in patients with idiopathic thrombocytopenic purpura. Thromb Res.

[12] Yu J, Heck S, Patel V, Levan J, Yu Y, Bussel JB, Yazdanbakhsh K (2008). Defective circulating CD25 regulatory T cells in patients with chronic immune thrombocytopenic purpura. Blood.

[13] Andersson PO, Stockelberg D, Jacobsson S, Wadenvik H (2000). A transforming growth factor-β1-mediated bystander immune suppression could be associated with remission of chronic idiopathic thrombocytopenic purpura. Ann Hematol.

[14] Nishimoto T, Kuwana M (2013). CD4+CD25+Foxp3+ regulatory T cells in the pathophysiology of immune thrombocytopenia. Semin Hematol.

[15] Mazzucco KL, Junior LM, Lemos NE, Wieck A, Pezzi A, Laureano AM, Amorin B, Valim V, Silla L, Daudt LE, Marostica PJ (2013). Assessment of regulatory T cells in childhood immune thrombocytopenic purpura. ISRN Hematol.

